# What Is the Optimal Radiation Technique for Esophageal Cancer? A Dosimetric Comparison of Four Techniques

**DOI:** 10.7759/cureus.2985

**Published:** 2018-07-16

**Authors:** Ziad Simon Fawaz, Suzanne Kazandjian, James M Tsui, Dr Slobodan Devic, Magali Lecavalier-Barsoum, Te Vuong, Sara Elakshar, Aurelie Garant, Isabelle Lavoie, Tamin M Niazi

**Affiliations:** 1 Radiation Oncology, McGill University Health Center, Montreal, CAN; 2 Radiation Oncology, McGill University/Sir Mortimer B. Davis Jewish General Hospital, Montreal, CAN; 3 Oncology/Radiation Oncology, Cedars Cancer Centre/McGill University Health Centre, Montreal, CAN; 4 Radiation Oncology, McGill University Health Centre, Montreal, CAN; 5 Radiation Oncology, McGill University/Jewish General Hospital, Montreal, CAN; 6 Oncology, McGill University Health Centre/Jewish General Hospital, Montreal , CAN

**Keywords:** esophageal, cancer, technique, conformal, dosimetric, imrt, vmat

## Abstract

Background

Esophageal cancer treatment requires large radiation fields due to the deep location of the esophagus in the mediastinum and the high incidence of radial spread. There is no optimal radiation technique to ensure appropriate target coverage and minimal dose to all normal structures.

Methods

Fifteen consecutive cases of locally advanced esophageal cancer treated with radical chemoradiation (CRT) were analyzed. The total prescribed dose was 50.4 Gy in 28 fractions. A total of 60 plans were generated for analysis, including four different methods for each case. Method 1 consisted of a four-field conformal technique; method 2 was a two-plan technique (antero-posterior (AP), postero-anterior (PA), two posterior oblique fields (RPO and LPO)); method 3 was a three-field conformal technique (AP, LPO, RPO); and method 4 was a volumetric modulated arc radiotherapy (VMAT) technique. Dose ratios were calculated using the minimum, maximum, mean, and median doses of methods 2-4 over the dose of method 1. Ratios for the planning target volume (PTV) and to surrounding organs were analyzed.

Results

The mean PTV dose ratio ranged from 0.994 to 1.048 (SD = 0.01) representing an adequate target coverage for all techniques based on an analysis of variance (ANOVA). For the lungs, method 2 had the lowest lung V20 with a ratio of 0.861 (SD = 0.12), whereas method 3 had the highest with 1.644 (SD = 0.14). For the heart, method 3 had the lowest heart V40 with a mean dose ratio of 0.807 (SD = 0.09), whereas method 2 had the highest with 1.160 (SD = 0.11). For the liver, method 2 had the lowest V30 with a mean ratio of 0.857 (SD = 0.1) whereas method 4 had the highest with 1.672 (SD = 0.48). For the spinal cord, method 3 had the lowest mean dose ratio of 0.559 (SD = 0.09) whereas method 2 had the highest with 1.094 (SD = 0.04).

Conclusion

The four radiation techniques for esophageal cancer treatment were appropriate for target coverage. Method 2 had the most organ-sparing effect for the lungs and liver, and method 3 for the heart and spinal cord. VMAT did not add any significant sparing. A case-by-case decision should be made based on the patient’s comorbidities.

## Introduction

Esophageal cancer is an aggressive disease with half of the patients presenting at a locally advanced stage [1-2]. Locoregional and distant recurrence rates are high, while the five-year overall survival is low, ranging from 30% to 40% [3]. Treatment of locally advanced esophageal cancer requires a multimodal approach that includes neoadjuvant chemoradiation (CRT) and resection for resectable disease, and radical concurrent CRT for unresectable disease. In both settings, radiation treatment volumes are large to account for the well-described tendency of esophageal cancer to extend submucosally in the longitudinal direction over a considerable distance [4]. These large treatment volumes are associated with higher toxicities and call for judicious radiation planning.

Toxicities of grade 3 or more are reported in up to 15% of the patients during this treatment, which can lead to a possible delay in treatment [5-6]. These challenges raise the question of what is the optimal radiation technique that limits toxicities while delivering the appropriate target coverage. Different techniques have been proposed including three-dimensional conformal radiotherapy (3D-CRT), fixed-field intensity-modulated radiotherapy (IMRT), volumetric modulated arc radiotherapy (VMAT), and helical tomotherapy [7-13]. However, there is still no clear consensus on a preferred technique.

Thus, we conducted a review of radiation treatment plans for locally advanced esophageal cancer treated at our institution. Our goal was to compare four commonly used radiation techniques in terms of target coverage and dose to the normal structures.

## Materials and methods

Fifteen cases of locally advanced esophageal cancer that were treated with radical CRT were included. The total prescribed dose was 50.4 Gy in 1.8 Gy per fraction. Patient characteristics including the age at diagnosis, gender, smoking status, histology, location of the tumor, size of the tumor, nodal status, setting, and date of treatment were gathered (Table 1).

**Table 1:** Patient characteristics SCC: Squamous cell carcinoma

Characteristic	Mean (n = 15)
Age (in years)	65 (range: 20-85)
Gender	73% male 27% female
Smoking	33% smoker 27% non-smoker 40% not available
Histology	73% SCC 20% adenocarcinoma 7% sarcoma
Location of tumor	13% upper third 20% middle third 67% lower third
Size (in cm)	5 (range: 2-12)
Nodal status	53% N0 47% N+
Setting	93% radical 7% adjuvant
Date of treatment	Feb 2006 until Aug 2010

The initial plan for all the cases was done with method 1: four fields 3D-CRT, including one anterior-posterior field (AP) field, one posterior-anterior field (PA) field, and two posterior obliques (RPO and LPO). This technique was the standard at our institution at the time of the analysis. Using the dose ratios, the three other planning techniques were compared. The dose ratios were obtained using the minimum, maximum, mean, and median dose to the target and to the normal surrounding structures of the three comparison methods over method 1.

Each case was reviewed and planned using all four methods for the 15 cases (Table 2), thus generating a total of 60 plans for analysis. Method 2 included AP/PA fields for plan 1, and AP, RPO, and LPO for plan 2. Method 3 consisted of a one-plan three-field technique (3F): AP/LPO/RPO. Method 4 consisted of a volumetric modulated arc radiotherapy plan (VMAT). 

**Table 2:** Methods used for the dosimetric comparison 3D-CRT (three-dimensional conformal radiation therapy), AP (anterior-posterior), PA (posterior-anterior), RPO (right posterior oblique), LPO (left posterior oblique), VMAT (volumetric modulated arc radiotherapy)

Method 1 (reference for comparison)	4 fields 3D-CRT: 1 AP, 1 PA, and 2 posterior oblique fields (RPO, LPO)
Method 2	Plan 1 (AP/PA field) and Plan 2 (AP field and 2 posterior oblique fields RPO/LPO)
Method 3	3 fields 3D-CRT (AP, RPO, LPO)
Method 4	VMAT

The minimum, maximum, mean, and median dose to the planning target volume (PTV) for each method was calculated and compared to method 1 by taking the dose ratio. A ratio larger than 1 represents a larger dose delivered as compared to method 1. Conversely, a ratio less than 1 indicates a lesser dose delivered as compared to method 1. The organs at risk included the lungs, the heart, the liver, and the spinal cord. The mean and median doses were calculated for all the organs. The V20, V40, and V30 doses were calculated for the lungs, heart, and liver respectively. A one-way analysis of variance (ANOVA) was then conducted to test for the differences between the dose ratios.

## Results

Target coverage

Methods 1 to 3, all the 3D-CRT planning techniques, produced acceptable dose distributions with doses within -5% and +7% of the prescribed dose as recommended by the International Commission on Radiation Units and Measurements (ICRU) Report 50 (Table 3).

**Table 3:** Range of doses (in Gy) to the target and the OAR PTV (planning target volume), VMAT (volumetric modulated arc therapy)

Volume	Parameter	Method 1 (Four fields)	Method 2 (Two plans)	Method 3 (Three fields)	Method 4 (VMAT)
PTV coverage	Mean dose	43.2 - 51.9	43.2 - 52.0	44.1 - 51.7	52.6 - 53.2
	Maximum dose	51.3 - 53.7	51.2 - 53.8	51.1 - 54.1	55.2 - 56.3
Liver	Mean dose	0.04 - 18.1	0.04 - 16.6	0.05 - 23.8	0.08 - 14.2
	V30	0.05 - 21.9	0.04 - 16.1	0.11 - 36.3	0.10 - 20.2
Lungs	Mean dose	4.81 - 16.4	4.72 - 15.6	6.62 - 21.6	5.87 - 16.9
	V20	7.74 - 24.0	7.50 - 26.8	14.0 - 34.8	12.7 - 22.1
Spinal cord	Mean dose	10.7 - 25.7	11.0 - 28.6	5.22 - 13.6	6.83 - 21.6
	Maximum dose	34.1 - 48.1	39.0 - 50.6	16.6 - 47.1	23.7 - 45.1
Heart	Mean dose	0.26 - 40.9	0.25 - 44.0	0.26 - 38.3	0.58 - 32.3
	V40	0.25 - 45.2	0.23 - 48.6	0.24 - 44.7	0.56 - 31.8

**Figure 1:**
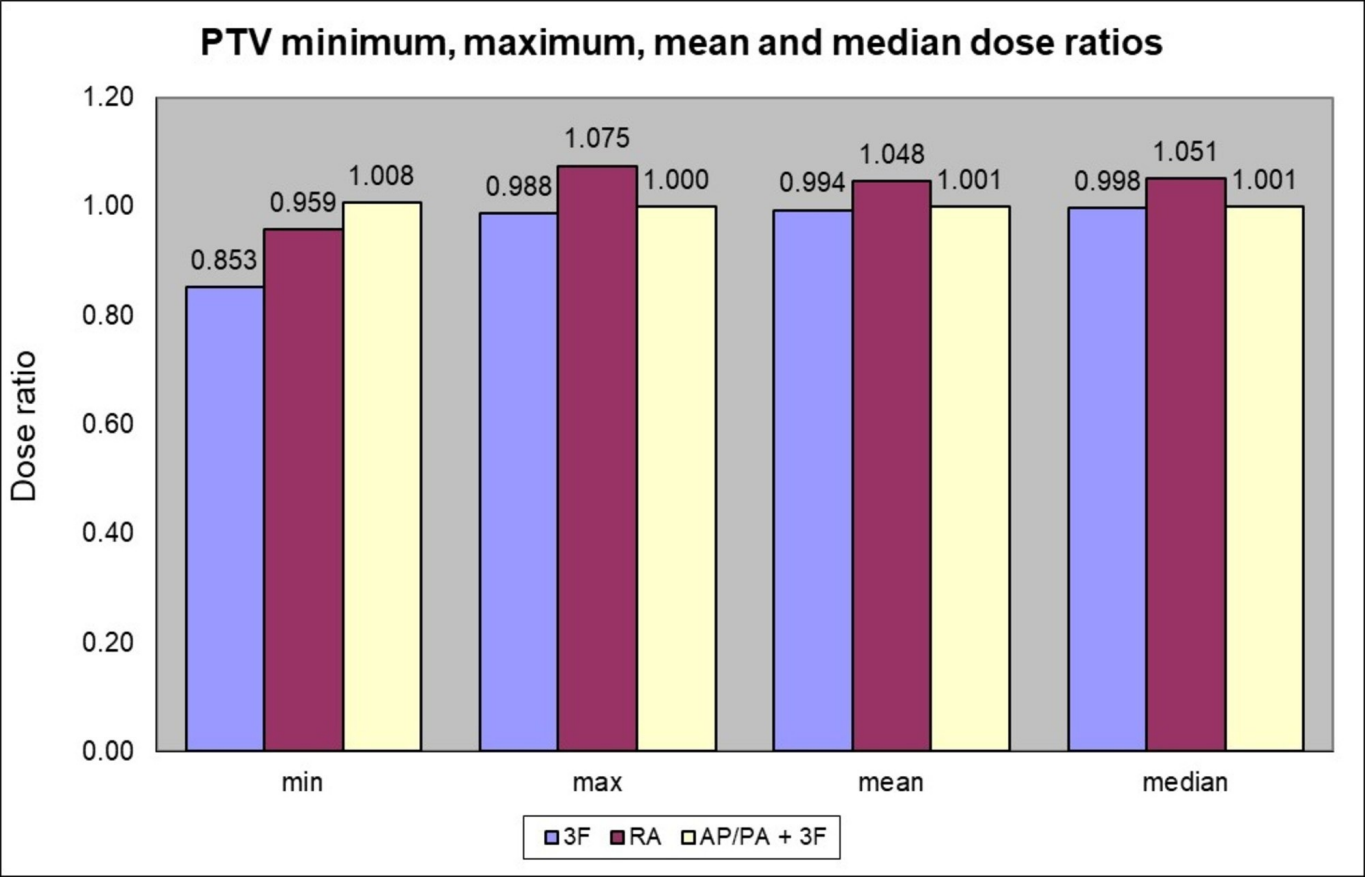
PTV minimum, maximum, mean and median dose ratios according to the method used compared to 4 fields 3D-CRT (method 1) PTV (planning target volume), 3F (3 fields), RA (RapidArc VMAT), AP/PA (anterior-posterior/posterior-anterior)

The PTV doses were similar and adequate for all the techniques with a mean dose ratio value of 0.994 to 1.048 (SD = 0.01). Higher maximum dose with the VMAT (method 4) occurred as shown in Figure 1. The PTV maximum dose in the latter was 56.3 Gy as compared to 53.7 Gy for the reference method 1 of the four-fields 3D-CRT.

Organs at risk

Lungs

Method 2 had the lowest lung V20 with a dose ratio of 0.861 (SD = 0.12). Method 3 (three-fields) had the highest dose ratio with 1.644 (SD = 0.14), and method 4 (VMAT) had a ratio of 1.150 (SD = 0.25). The mean dose ratio for the lungs was the lowest for method 2 with a value of 0.9407 (SD = 0.0343). Both method 3 and 4 had higher mean dose ratios with 1.393 (SD = 0.0896) and 1.1740 (SD = 0.1866) respectively.

**Figure 2:**
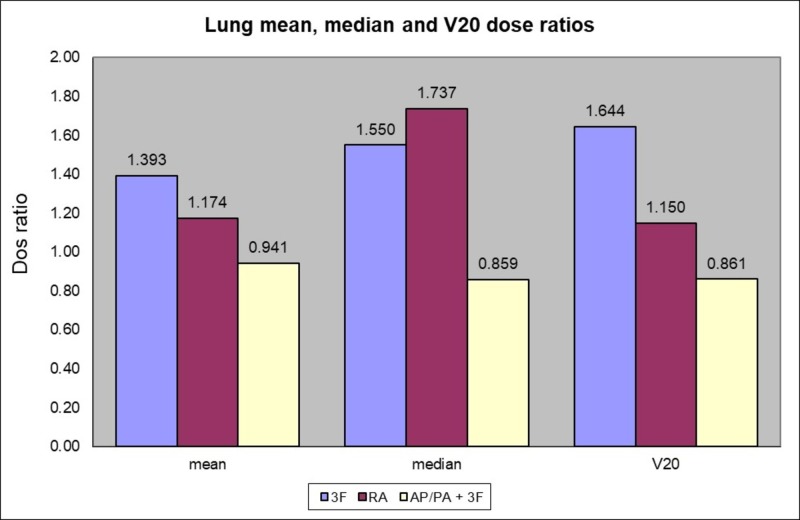
Lung mean, median and V20 dose ratios according to the method used compared to 4 fields 3D-CRT (method 1) 3F (three fields), RA (RapidArc VMAT), AP/PA (anterior-posterior/posterior-anterior)

The one-way ANOVA analysis showed that the method used had a significant effect on the lung V20 mean dose at the p < 0.05 level [F (3, 56) = 72.3190, p = 0.00001]. Post hoc comparisons using the Tukey honestly significant difference (HSD) test indicated that the mean score for method 1 was lower than the mean score for method 3 (p = 0.00001) and method 4 (p = 0.05), and that method 2 was significantly lower than method 3 (p = 0.00001) and method 4 (p = 0.00001), and that method 3 was significantly lower than method 4 (p = 0.00001). Taken together, these results suggest that methods 1 and 2 provided a significant sparing of the lung V20 dose as compared to method 3 and 4 (Figure 2).

Heart

Method 2 had the highest heart dose ratios with a V40 dose ratio of 1.160 (SD = 0.11). The three-field technique of method 3 had the lowest heart V40 with a mean dose ratio of 0.807 (SD = 0.09). Method 4 (VMAT) also had a low dose ratio of 0.824 (SD = 0.45).

**Figure 3:**
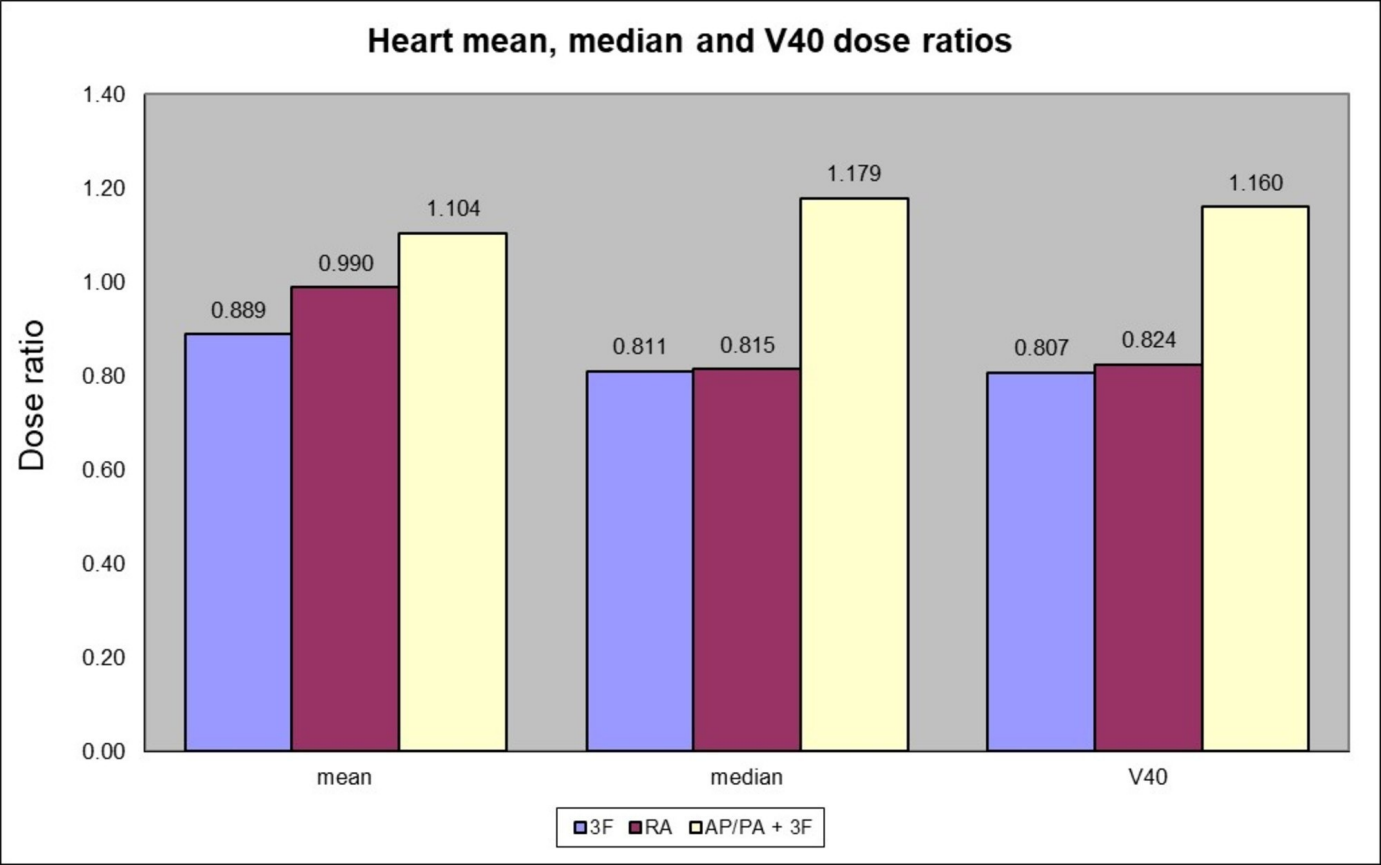
Heart mean, median and V40 dose ratios according to the method used compared to 4 fields 3D-CRT (method 1) 3F (three fields), RA (RapidArc VMAT), AP/PA (anterior-posterior/posterior-anterior)

The one-way ANOVA analysis showed that the method used had a significant effect on the heart V40 mean dose at the p < 0.05 level [F(3, 56) = 7.4421, p = 0.0003]. Post hoc comparisons using the Tukey HSD test indicated that the mean scores for method 3 (p = 0.0008) and method 4 (p = 0.0015) were significantly lower than method 2, but not significantly lower than method 1. Taken together, these results suggest that method 3 and 4 provided a significant sparing of the V40 heart dose as compared to method 2 (Figure 3).

Liver

VMAT (method 4) had the highest median and V30 dose to the liver with a ratio of 1.672 (SD = 0.48). Method 2 had the lowest liver V30 with a mean ratio of 0.857 (SD = 0.1), whereas method 3 had a mean ratio of 1.349 (SD = 0.38) for the liver V30.

**Figure 4:**
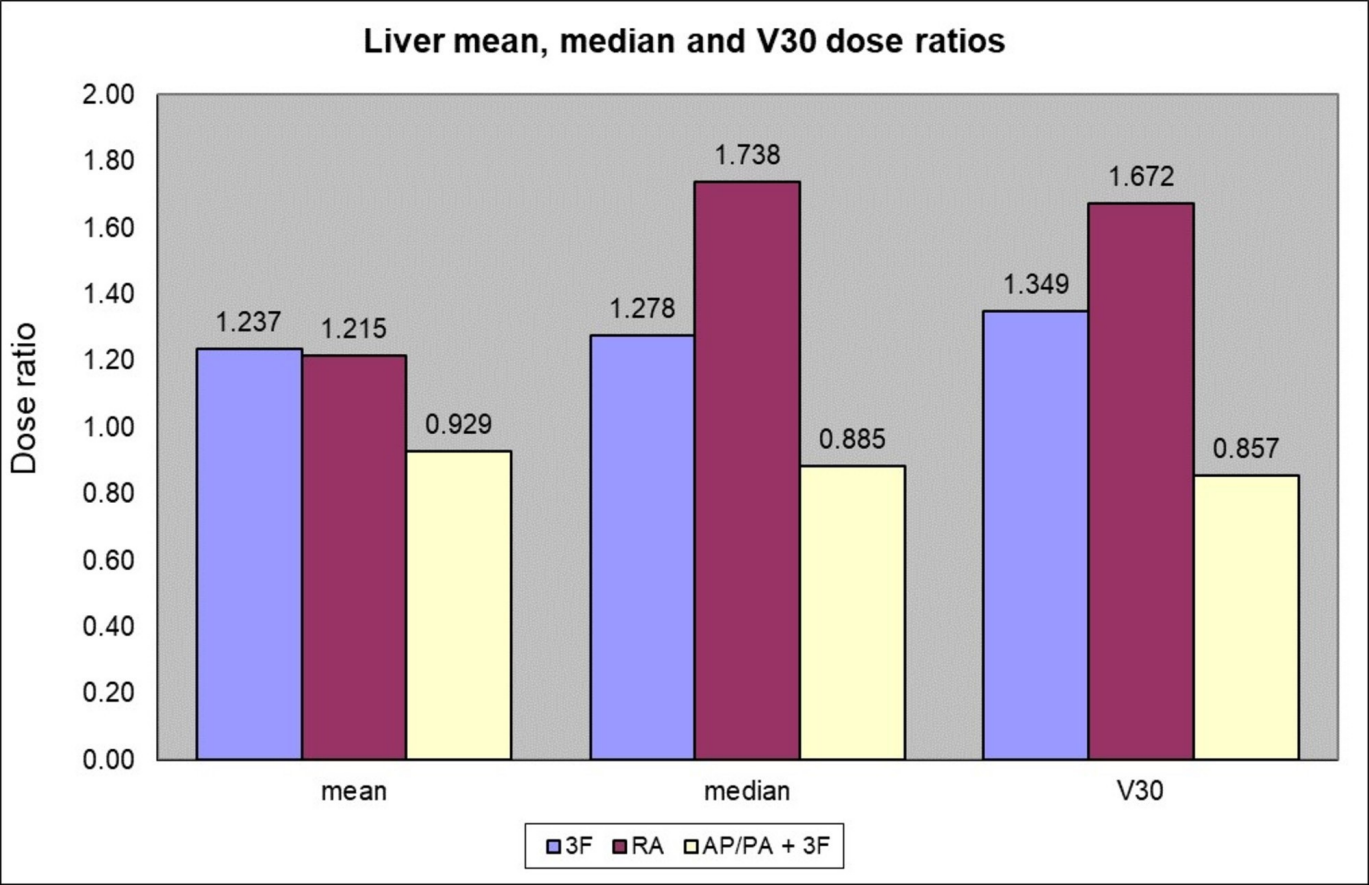
Liver mean, median and V30 dose ratios according to the method used compared to 4 fields 3D-CRT (method 1) 3F (three fields), RA (RapidArc VMAT), AP/PA (anterior-posterior/posterior-anterior)

The one-way ANOVA analysis showed that the method used had a significant effect on the liver V30 mean dose at the p < 0.05 level [F(3, 56) = 20.8425, p = 0.00001]. Post hoc comparisons using the Tukey HSD test indicated that the mean score for method 1 was significantly different than method 3 (p = 0.0164) and method 4 (p = 0.00001), and that method 2 was significantly different than method 3 (p = 0.0003) and method 4 (p = 0.00001), and that method 3 was significantly different than method 4 (p = 0.0301). Taken together, these results suggest that methods 1 and 2 provided a significant sparing of the V30 liver dose compared to methods 3 and 4 (Figure 4).

Spinal cord

Method 2 had the highest cord dose ratios with a mean dose ratio at 1.094 (SD = 0.04). For method 3, the spinal cord, the mean dose was the lowest with a dose ratio of 0.559 (SD = 0.09). Method 4 had a ratio of 0.759 (SD = 0.17). The maximum absolute doses for methods 1, 2, 3, and 4 were 48 Gy, 50.6 Gy, 47 Gy and 45 Gy respectively.

**Figure 5:**
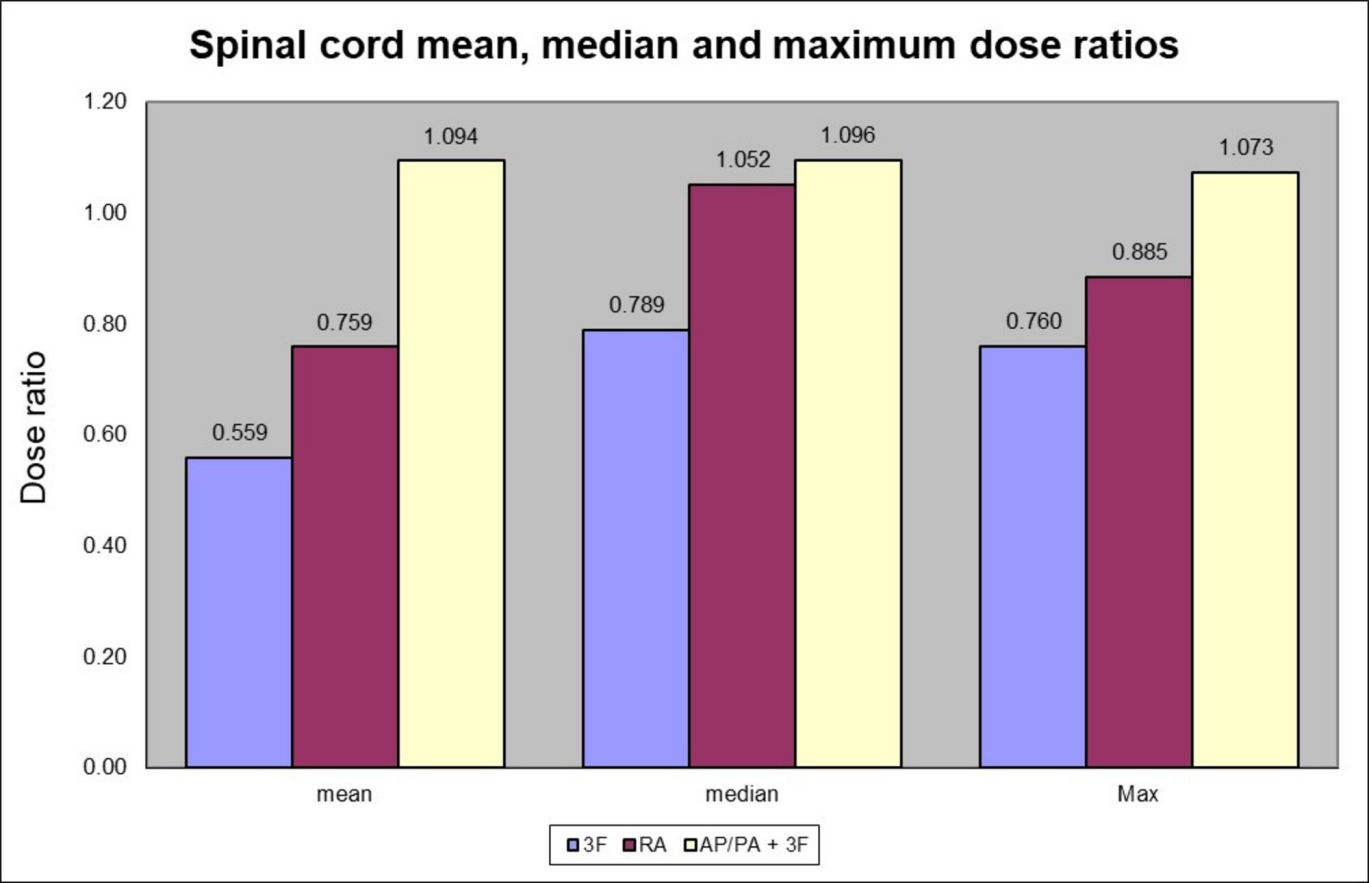
Spinal cord mean, median and maximum dose ratios according to the method used compared to 4 fields 3D-CRT (method 1) 3F (three fields), RA (RapidArc VMAT), AP/PA (anterior-posterior/posterior-anterior)

The one-way ANOVA analysis showed a significant effect for the method used for the spinal cord on the mean dose at the p < 0.05 level [F(3, 56) = 90.4196, p = 0.00001]. Post hoc comparisons using the Tukey HSD test indicated that the mean score for method 1 was significantly different than method 3 (p = 0.00001) and method 4 (p = 0.00001), and that method 2 was significantly different than method 3 (p = 0.00001) and method 4 (p = 0.00001), and that method 3 was significantly different than method 4 (p = 0.00001). Taken together, these results suggest that method 1 and 2 provide a significant sparing of the spinal cord mean dose compared to method 3 and 4 (Figure 5).

## Discussion

Recent technological advances have led to higher access and usage of more conformal radiation techniques including IMRT. The largest population-based review of 474,533 patients treated with radiation shows that the usage rates of IMRT increased from 2.1% in 2002 to 21.9% in 2012 for all tumor sites combined [14]. This has led to the adoption of more conformal techniques in some institutions without the data supporting a dosimetric advantage in terms of target coverage and sparing of the organs at risk (OAR).

In esophageal cancer, different treatment techniques, including three-dimensional conformal techniques, different fixed-field IMRT, and VMAT have been compared in the literature. In order to conduct appropriate comparisons and to ensure a consistent contouring of the clinical target volume, Wu et al. (2015) published an expert consensus contouring guideline for IMRT in the esophageal and gastroesophageal junction cancers [15].

Using consensus target contours, Allaveisi (2017) compared the four-field box and field-in-field techniques in terms of conformity, homogeneity, mean dose, and maximum dose [16]. The field-in-field technique resulted in a more homogenous dose distribution with a similar conformality. However, this comparison was done between two three-dimensional conformal techniques only. Fu et al. (2017) have compared plans including four, five, and seven beams of fixed-field IMRT [17]. Their findings suggested a similar maximum dose, mean dose, and conformity index for the three plans with similar coverage. However, the mean V5, V13, V20 and mean lung dose were significantly lower in the four-field plan compared to the five and seven field plans.

Three studies have compared VMAT versus fixed-field IMRT in the treatment of esophageal carcinoma. One study compared a VMAT technique with the conventional fixed-field IMRT plans in terms of PTV coverage and sparing of the OARs in thirteen patients [18]. The target coverage, the PTV D99, PTV mean, and PTV D95 were equivalent, but the maximum dose and the lung V20 were significantly lower in VMAT when compared to IMRT. The treatment time delivery was also reduced by up to 55% with VMAT.

Another study compared VMAT, using single and double arcs, with fixed-field IMRT using five, seven, and nine fields [19]. The study included 20 patients and was based on an analysis of the PTV coverage and doses to the OARs. Both the IMRT and VMAT plans provided an appropriate dose coverage to the PTV, but VMAT resulted in a higher conformity and lower lung V30 than the IMRT plans. The doses to the heart, including V30, V40, and V50, were lower with VMAT when compared to IMRT.

In a more recent study, Lin et al. (2014) assessed nine cases of middle-thoracic esophageal cancer and compared the PTV coverage, dose to the lungs, and delivery time with VMAT versus fixed-field IMRT. The VMAT plans provided superior PTV coverage, whereas the IMRT plans provided higher dose homogeneity. The V20 for the lungs was lower with VMAT plans, but V5 and V10 were lower with IMRT plans. VMAT also resulted in significantly shorter delivery time, necessitating only half of the time required for IMRT plans [20]

However, all of these dosimetric comparisons were done with either three-dimensional conformal techniques or IMRT techniques and there has not been a comparison of three-dimensional conformal techniques and VMAT for the same esophageal cases. Our paper is the first to report a comparison between these treatment methods. Each one of the techniques did better with the sparing of one or two organs, but at the expense of other organs. For the lungs and the liver, method 2, which is delivered in two plans, had the least mean, median, and maximum, or V40 dose ratios. However, it resulted in a higher heart mean, median, and V40 dose ratios due to the anterior-posterior, posterior-anterior, and one of the posterior oblique fields exiting through the hearts. It has the benefit of decreasing the mean, median, and V20 dose to the lungs, at the expense of the heart doses.

For the heart and the spinal cord, it was the three-field method 3 technique that was the least toxic. Method 4 (VMAT) did not provide any additional organ-sparing effect. Nonetheless, IMRT techniques such as VMAT use a computer-based optimization software that can ensure consistent planning from one case to the next. Three-dimensional techniques are dosimetrist-dependent and require considerable levels of experience to reproduce adequate plans.

Our findings suggest that there is no unique optimal radiation technique for the treatment of esophageal cancer, and that a case-by-case decision is needed to adapt the radiation technique based on the patient’s comorbidities. For example, a patient with multiple cardiac comorbidities would benefit from planning with method 3 which reduces the dose to the heart whereas a patient with multiple lung comorbidities could be better treated using method 2 which reduces the dose to the lungs. Further studies should aim at comparing new techniques as well as hybrid techniques [21] or simulated integrated boost [22].

## Conclusions

The 3D conformal and VMAT radiation methods are adequate techniques to deliver appropriate doses to the target. Method 2 (two-plans with anterior-posterior, posterior-anterior, and posterior oblique fields) had the most organ-sparing effect for the lungs and the liver, and method 3 (three fields) for the heart and the spinal cord. VMAT did not add significant sparing to the normal structures. Our data suggested there is no dosimetry technique that is optimal for all circumstances and therefore, the esophageal radiation treatment technique should be a case-by-case decision to adapt the method based on the patient's comorbidities.

## References

[1] Siegel R, Naishadham D, Jemal A (2012). Cancer statistics, 2012. CA Cancer J Clin.

[2] Rustgi A, El-Serag H (2014). Esophageal carcinoma (review). N Engl J Med.

[3] Shridhar R, Almhanna K, Meredith KL, Matthew C (2013). Radiation therapy and esophageal cancer. SAGE Open.

[4] Miller C (1962). Carcinoma of thoracic oesophagus and cardia: a review of 405 cases. Br J Surg.

[5] Emami B, Lyman J, Brown A (1991). Tolerance of normal tissue to therapeutic irradiation. Int J Radiat Oncol Biol Phys.

[6] Wei X, Liu HH, Tucker SL (2008). Risk factors for pericardial effusion in inoperable esophageal cancer patients treated with definitive chemoradiation therapy. Int J Radiat Oncol Biol Phys.

[7] Wu VW, Sham JS, Kwong DL (2004). Inverse planning in three-dimensional conformal and intensity-modulated radiotherapy of mid-thoracic oesophageal cancer. Br J Radiol.

[8] Chandra A, Guerrero TM, Liu HH (2005). Feasibility of using intensity-modulated radiotherapy to improve lung sparing in treatment planning for distal esophageal cancer. Radiother Oncol.

[9] Chen YJ, Liu A, Han C (2007). Helical tomotherapy for radiotherapy in esophageal cancer: a preferred plan with better conformal target coverage and more homogeneous dose distribution. Med Dosim.

[10] Hong TS, Crowley EM, Killoran J, Mamon HJ (2007). Considerations in treatment planning for esophageal cancer. Semin Radiat Oncol.

[11] Yang B, Zhu L, Cheng H, Li Q, Zhang Y, Zhao Y (2009). Dosimetric comparison between intensity-modulated radiotherapy and conformal radiotherapy for upper thoracic esophageal carcinoma. Precis Radiat Oncol.

[12] Van Benthuysen L, Hales L, Podgorsak MB (2011). Volumetric modulated arc therapy vs. IMRT for the treatment of distal esophageal cancer. Med Dosim.

[13] Vivekanandan N, Sriram P, Kumar SA, Bhuvaneswari N, Kamalakannan S (2012). Volumetric modulated arc radiotherapy for esophageal cancer. Med Dosim.

[14] Waddle MR, Sio TT, Van Houten HK (2017). Photon and proton radiation therapy utilization in a population of more than 100 million commercially insured patients. Int J Radiat Oncol Biol Phys.

[15] Wu AJ, Bosch WR, Chang DT (2015). Expert consensus contouring guidelines for intensity modulated radiation therapy in esophageal and gastroesophageal junction cancer. Int J Radiat Oncol Biol Phys.

[16] Allaveisi F, Moghadam AN (2017). Comparison between the four-field box and field-in-field techniques for conformal radiotherapy of the esophagus using dose-volume histograms and normal tissue complication probabilities. Jpn J Radiol.

[17] Fu Y, Deng M, Zhou X (2017). Dosimetric effect of beam arrangement for intensity-modulated radiation therapy in the treatment of upper thoracic esophageal carcinoma. Med Dosim.

[18] Abbas AS, Moseley D, Kassam Z, Kim SM, Cho C (2013). Volumetric-modulated arc therapy for the treatment of a large planning target volume in thoracic esophageal cancer. Med Phys.

[19] Yin L, Wu H, Gong J (2012). Volumetric-modulated arc therapy vs. c-IMRT in esophageal cancer: a treatment planning comparison. World J Gastroenterol.

[20] Lin CY, Huang WY, Jen YM (2014). Dosimetric and efficiency comparison of high-dose radiotherapy for esophageal cancer: volumetric modulated arc therapy versus fixed-field intensity-modulated radiotherapy. Dis Esophagus.

[21] Mayo CS, Urie MM, Fitzgerald TJ (2008). Hybrid IMRT for treatment of cancers of the lung and esophagus. Int J Radiat Oncol Biol Phys.

[22] Fu WH, Wang LH, Zhou ZM (2004). Comparison of conformal and intensity-modulated techniques for simultaneous integrated boost radiotherapy of upper esophageal carcinoma. World J Gastroenterol.

